# TGF-Beta Receptor II Is Critical for Osteogenic Progenitor Cell Proliferation and Differentiation During Postnatal Alveolar Bone Formation

**DOI:** 10.3389/fphys.2021.721775

**Published:** 2021-09-24

**Authors:** Chunmei Xu, Xudong Xie, Hu Zhao, Yafei Wu, Jun Wang, Jian Q. Feng

**Affiliations:** ^1^State Key Laboratory of Oral Diseases, National Clinical Research Center for Oral Diseases, Department of Periodontics, West China Hospital of Stomatology, Sichuan University, Chengdu, China; ^2^Department of Biomedical Sciences, College of Dentistry, Texas A&M University, Dallas, TX, United States; ^3^Department of Comprehensive Dentistry, College of Dentistry, Texas A&M University, Dallas, TX, United States

**Keywords:** mandible, alveolar bone, GLI1, osteoprogenitor, osteoblast, TGFβR2

## Abstract

Transforming growth factor beta (TGFβ) signaling plays an important role during osteogenesis. However, most research in this area focuses on cortical and trabecular bone, whereas alveolar bone is largely overlooked. To address the role of TGFβR2 (the key receptor for TGFβ signaling) during postnatal alveolar bone development, we conditionally deleted *Tgf*β*r2* in early mesenchymal progenitors by crossing *Gli1-Cre*^*ERT*2^; *Tgf*β*r2*^*flox*/*flox*^; *R26R*^*tdTomato*^ mice (named early cKO) or in osteoblasts by crossing *3.2kb Col1-Cre*^*ERT*2^*; Tgf*β*r2* ^*flox*/*flox*^*; R26R*^*tdTomato*^ mice (named late cKO). Both cKO lines were induced at postnatal day 5 (P5) and mice were harvested at P28. Compared to the control littermates, early cKO mice exhibited significant reduction in alveolar bone mass and bone mineral density, with drastic defects in the periodontal ligament (PDL); conversely, the late cKO mice displayed very minor changes in alveolar bone. Mechanism studies showed a significant reduction in PCNA+ PDL cell numbers and OSX+ alveolar bone cell numbers, as well as disorganized PDL fibers with a great reduction in periostin (the most abundant extracellular matrix protein) on both mRNA and protein levels. We also showed a drastic reduction in β-catenin in the early cKO PDL and a great increase in SOST (a potent inhibitor of Wnt signaling). Based on these findings, we conclude that TGFβ signaling plays critical roles during early alveolar bone formation via the promotion of PDL mesenchymal progenitor proliferation and differentiation mechanisms.

## Introduction

The skeleton is formed by two osteogenic processes: endochondral ossification and intramembranous ossification (Long, 2011). Similarly, mandibular bone is considered to be formed by the same processes, with the mandibular body formed through intramembranous ossification and the mandibular ramus built via endochondral ossification (Hinton et al., 2015, 2017; Jing et al., 2015). On the other hand, we have recently begun to gain knowledge on a third type of ossification process within alveolar bone, which holds teeth. Cell lineage tracing studies showed that periodontal ligament (PDL) progenitor cells contribute to alveolar bone formation and regeneration (Ren et al., 2015; Hosoya et al., 2020; Men et al., 2020). This occurrence is distinct from that of other cell sources such as periosteum for the mandible body or chondrocytes for the mandible ramus (Hinton et al., 2015, 2017; Jing et al., 2015). Importantly, alveolar bone displayed a much higher bone formation rate than other types of bones (Ren et al., 2015).

Periodontitis, the most common disorder known to mankind, particularly affects alveolar bone. The advanced form of this condition results in loss of surrounding soft tissue and bone, leading to tooth loss in adults. This severe result was found in 10–15% of adults in population studies (Fox, 1992; Douglass and Fox, 1993; Fox et al., 1994). In addition, several pieces of evidence support a two-way relationship between periodontitis and diabetes (i.e., diabetes increases the risk for periodontitis, while periodontitis negatively disturbs glycemic control) (Hallmon and Mealey, 1992; Khader et al., 2006; Mealey and Ocampo, 2007; Salvi et al., 2008; Chavarry et al., 2009; Preshaw et al., 2012). Research has also demonstrated a close link between osteoporosis and periodontitis (Wang and McCauley, 2016). Experts have long aimed to develop effective treatment methods for the bone loss in these diseases (Pihlstrom et al., 2005). Thus, understanding the mechanism by which alveolar bone formation is regulated will facilitate future drug development.

It has been known that TGFβ signaling plays critical roles in intramembranous bone formation. These roles include bone development and fracture healing processes (Oka et al., 2007; Seo and Serra, 2007, 2009; Tang and Alliston, 2013; Wu et al., 2016; Xia et al., 2020) as well as regulation for the differentiation of osteogenic cells and extracellular matrix synthesis (Tang and Alliston, 2013; Peters et al., 2017).

TGFβR2 is required for the proliferation and differentiation of osteogenic progenitors from embryonic stages (Sasaki et al., 2006; Oka et al., 2007; Seo and Serra, 2009; Chen et al., 2012; Abou-Ezzi et al., 2019). Deletion of *Tgf*β*r2* driven by *Prx1-Cre* in the mesenchymal cells led to severe skeletal phenotypes characterized by short limbs and absent parietal bones as well as frontal bone at E15.5 (Seo and Serra, 2007). Oka et al. also reported reduced cell proliferation activity in mandibles of *Tgf*β*r2*^*flox*/*flox*^; *Wnt1-Cre* mice at E13.5 (Oka et al., 2007). In addition, TGFβ signaling plays a critical role in osteoblast lineage cells after birth (Meng et al., 2018). Disturbance of TGFβ signaling leads to severe defects in postnatal skeletal development (Peters et al., 2017; Corps et al., 2021). However, there is a debate regarding the exact role of TGFβ signaling in a specific population of cells. For example, Peters et al. reported disturbed differentiation of osteoblasts and reduced bone mass in long bone when *Tgf*β*r2* was removed in OSX+ bone cells (Peters et al., 2017). Abou-Ezzi et al. confirmed similar defects in trabecular bone and cortical bone (Abou-Ezzi et al., 2019). However, a drastic increase in trabecular bone mass was observed when *Tgf*β*r2* was conditionally deleted in OCN+ bone cells (Qiu et al., 2010). Few studies have focused on alveolar bone phenotypes, which require further investigation, especially during postnatal bone formation (Sasaki et al., 2006; Kim et al., 2015).

Given the limitations of non-inducible Cre transgenic mice in early studies and differing results from different studies, we intended to assess the postnatal effects of TGFβ signaling in alveolar bone development using inducible Cre mouse lines. Specifically, we included Gli1 (a marker for mesenchymal progenitor cells in various tissues including PDL and alveolar bone) to target the early progenitor cells (Kitaura et al., 2014; Feng et al., 2017; Shi et al., 2017; Hosoya et al., 2020; Liu et al., 2020; Men et al., 2020; Yi et al., 2021); 3.2 kb Col1 was used to target osteoblasts (Rossert et al., 1995; Qin et al., 2019). Our findings revealed a key role in TGFβ signaling during early postnatal alveolar bone development.

## Materials and Methods

### Breeding Transgenic Mice

All experimental protocols followed ARRIVE (Animal Research Reporting of *in vivo* Experiments) guidelines and were approved by the Animal Care and Use Committees (IACUC) at Sichuan University West China School of Stomatology and Texas A&M University College of Dentistry.

All mice (background: C57BL/6J) were housed in a temperature-controlled environment with 12-h light/dark cycles. To induce disrupted TGFβ signaling among osteogenic cells at different stages, we generated conditional *Tgf*β*r2* knockout mice (*Tgf*β*r2* cKO) driven by *Gli1-Cre*^*ERT*2^ (Ahn and Joyner, 2004) and the *3.2 kb Col1-Cre*^*ERT*2^ transgene, respectively (Rossert et al., 1995). The *Gli1-Cre*^*ERT*2/+^ mice were crossed with *R26R*^*tdTomato*/+^ reporter mice (stock number: 007905 from Jackson Laboratory) to trace the Cre activity. Next, we crossed the *Gli1-Cre*^*ERT*2/+^; *R26R*^*tdTomato*/+^ mice with *Tgf*β*r2*^*flox*/+^ mice (stock number: 012603 from Jackson Laboratory) to gain *Gli1-Cre*^*ERT*2/+^; *R26R*^*tdTomato*/+^; *Tgf*β*r2*^*flox*/+^ mice. Then, the *Gli1-Cre*^*ERT*2/+^; *R26R*^*tdTomato*/+^; *Tgf*β*r2*^*flox*/*flox*^ mice were obtained by crossing *Gli1-Cre*^*ERT*2/+^; *R26R*^*tdTomato*/+^; *Tgf*β*r2*^*flox*/+^ mice with the *Tgf*β*r2*^*flox*/+^ mice. The same strategy was then applied to generate *3.2 kb Col1-Cre*^*ERT*2/+^; *R26R*^*tdTomato*/+^; *Tgf*β*r2*^*flox*/*flox*^ mice. The genotypes of the mice were determined via a PCR analysis of genomic DNA extracted from tail biopsies (primer sequences are listed in Supplementary Table 1). Tamoxifen (75 mg/kg body weight) was prepared as previously described (Wang et al., 2020) and a one-time injection of the drug was administered at postnatal day 5 (P5) to both control mice (CTR: *Gli1-Cre*^*ERT*2/+^/*3.2 kb Col1-Cre*^*ERT*2/+^; *R26R*^*tdTomato*/+^) and cKO mice (*Gli1-Cre*^*ERT*2/+^/*3.2 kb Col1-Cre*^*ERT*2/+^; *R26R*^*tdTomato*/+^; *Tgf*β*r2*^*flox*/*flox*^). The animals were subsequently harvested at either P6 or P28. Mandibles were dissected and fixed in 4% paraformaldehyde (PFA) and decalcified in 10% ethylenediaminetetraacetic acid (EDTA), then stored at 4°C for future use.

### Histological Analysis and Immunostaining

Mandibles intended for histological staining were embedded in paraffin using standard histological procedures, then sectioned at 5-μm thickness for Masson's trichrome, Sirius red, and TRAP staining as previously reported (Wang et al., 2017). Samples for cell lineage tracing were dehydrated with 30% sucrose and embedded in OCT. Next, 10-μm-thick sections were prepared with a Leica cryostat equipped with Cryojane as previously reported (Xie et al., 2019). Immunostaining was then carried out as previously described (Wang et al., 2020) using the following primary antibodies: anti-OSX rabbit antibody (1:200, ab22552), anti-PERIOSTIN goat antibody (1:400, AF2955), anti-MEPE rabbit antibody (1:100, LF-155), anti-SOST goat antibody (1:100, AF1589), anti-PCNA rabbit antibody (1:100, Cst13110s). The secondary antibodies used for immunostaining: Goat anti-Rabbit IgG-Alexa Fluor 488 (1:200, Invitrogen); Rabbit anti-Goat IgG-Alexa Fluor 488 (1:200, Invitrogen); and Goat Anti-Rabbit IgG-unconjugated (1:100, Vector laboratories).

### Micro–Computed Tomography (μ-CT) and X-Ray Radiography Analysis

Micro-CT analysis by the Scanco μ-CT35 image system and X-ray radiography were performed as previously described (Wang et al., 2017).

### RNAscope Assay Procedures for RNA Detection

Mandibles were harvested and fixed in 10% formalin for 24 h at room temperature and then decalcified in 10% ethylenediaminetetraacetic acid (EDTA) for 3 weeks at 4°C. Well-decalcified samples were embedded in paraffin and cut according to previously mentioned standard histological procedures. 5-μm-thick sections were collected and an RNAscope assay was performed following the RNAscope®2.5 BROWN (Advanced Cell Diagnostics, 322300, 322310) for FFPE manufacturer protocol (Wang et al., 2012) with use of the β-catenin RNA probe (537601) and Periostin RNA probe (418581).

### Statistical Analysis

Statistical analyses were performed by an independent sample *t*-test for parametric analysis and the Mann-Whitney test was used for non-parametric analysis using SPSS 19.0 (SPSS Inc, Chicago, IL). A *P*-value < 0.05 was considered statistically significant.

## Results

### Removing TGFβ Signaling in Gli1+ Progenitors Resulted in Disturbance of Periodontal Homeostasis and Postnatal Alveolar Bone Loss Due to a Defect in PDL Progenitor Cells

**Figure 1:**
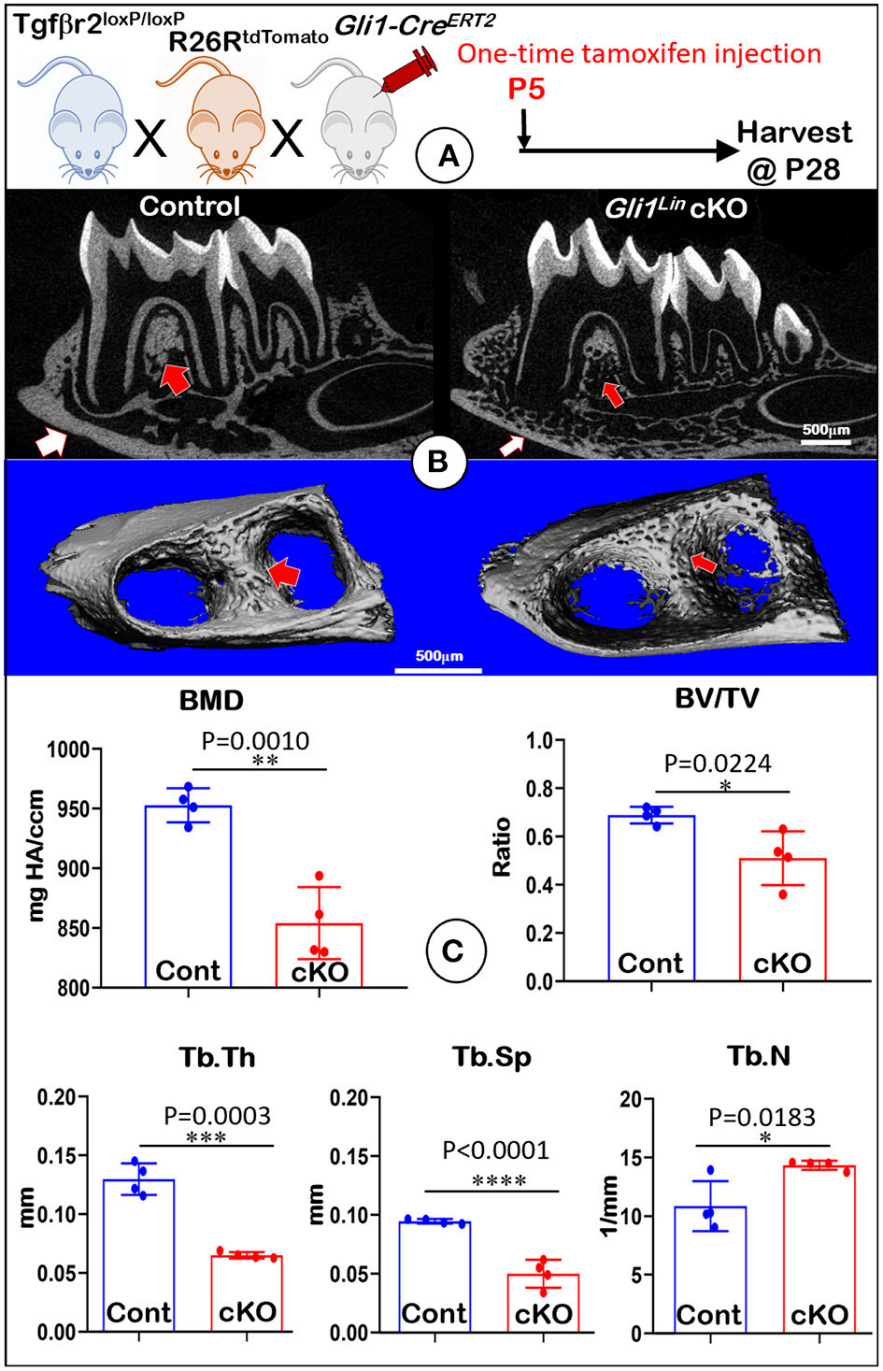
Micro-computed tomography (μCT) analyses showed defective mandibles from Gli1^Lin^ *Tgf*β*r2* conditional knockout (cKO) mice. **(A)** A schematic illustration of the generation of the *Gli1-Cre*^*ERT*2^ induced *Tgf*β*r2* cKO mice in the R26R^tdTomato^ tracing background. A one-time tamoxifen injection was administered at P5, and harvested at P28. **(B)** The cKO mice showed remarkable decrease of bone mass at both trabecular (red arrows) and cortical (white arrows) regions. **(C)** Quantitative data based on the trabecular bone showed a significant reduction in bone mineral density (BMD) (*upper left*), the bone volume fraction (BV/TV) (*upper right*), and trabecular thickness (Tb.Th) (*lower left*), and trabecular separation (Tb.Sp) (*lower middle*). Of note, there was significant increase in trabecular number (Tb. N) (*lower right*). *n* = 4, *P < *0.05*; **P < *0.01*; ***P < *0.001*; ****P < *0.0001*.

To address the postnatal effects of TGFβ signaling on osteogenic progenitors, we generated *Gli1*^*Lin*^ *Tgf*β*r2* cKO mice and performed a one-time injection of tamoxifen in both the control and cKO mice at P5. Mice were harvested at P28 (Figure 1A). Our representative X-ray images showed no obvious differences in hindlimbs between the control and cKO mice (Supplementary Figure 1). However, the sagittal section (Figure 1B, *upper panels*) and three-dimensional reconstruction (Figure 1B, *lower panels*) images displayed drastic bone loss in *Gli1*^*Lin*^ *Tgf*β*r2* cKO mandibles (Figure 1B, *right panels*). The overall cKO bone structure was porous in both alveolar bone and cortical bone (Figure 1B, *arrows*). The quantitative μCT data on alveolar bone (Figure 1C, *n* = 4) displayed a significant decrease in bone mineral density (BMD, *P* = 0.0010), bone volume fraction (BV/TV, *P* = 0.0224), trabecular bone thickness (Tb.Th, *P* = 0.0003), and trabecular bone separation (Tb.Sp, *P* < 0.0001). There was a moderate increase in trabecular bone numbers (Tb.N, *P* = 0.0183) in the cKO mice compared to control mice. These changes support the vital role of TGFβ signaling in control of postnatal alveolar bone formation.

**Figure 2:**
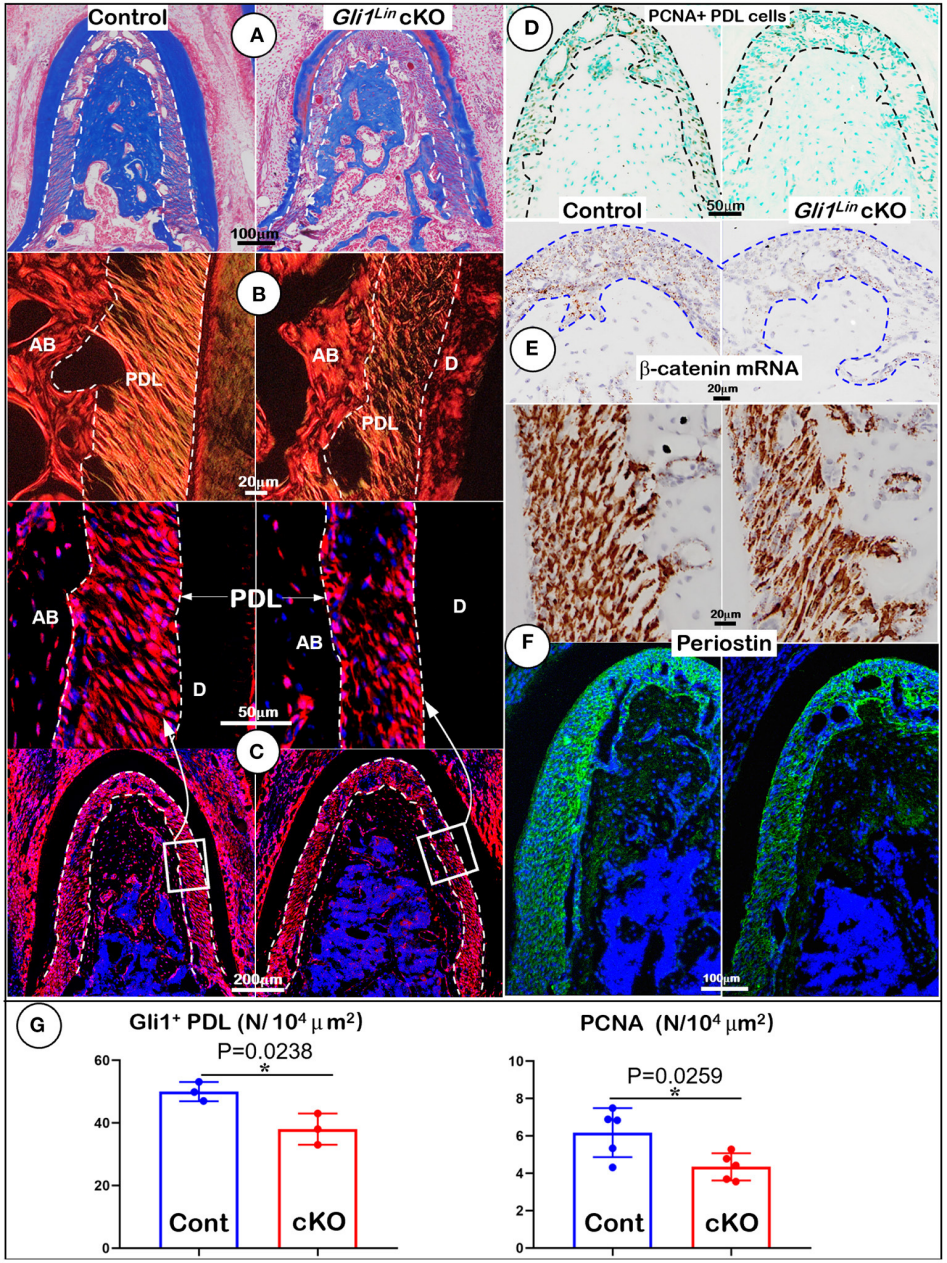
Morphological analyses of mandibles from Gli1^Lin^ *Tgf*β*r2* conditional knockout (cKO) mice at cellular levels. **(A)** Masson's Trichrome staining showed drastic reduction of alveolar bone mass in cKO mice (*right*); **(B)** Polarized light images displayed a decrease in periodontal ligament (PDL) with a disorganized collagen fiber distribution in cKO mice (*right*); **(C)** Gli1^Lin^ tracing images revealed pathological changes in the cKO PDL: irregular cell shape and a significant reduction in tdTomato+ PDL cell number compared to the spindle-shape PDL cells in the control (*upper right*); **(D)** The immunostaining images of PCNA showed a significant decline in proliferating cells in the cKO PDL; **(E)** The RNAscope images showed a drastic reduction in β-catenin mRNA level in the cKO PDL (*right*); and **(F)** a decrease of Periostin expression at both mRNA level (*upper right*) and protein level (*lower right*). **(G)** Quantitative analyses of the number of tdTomato+ PDL cells (*left*) and PCNA expressing cells in PDL (*right*). *n* = 3–5, *P < *0.05*. AB, alveolar bone; PDL, periodontal ligament; D, dentin.

To further address the impact of TGFβ signaling on osteogenesis at cellular levels, we first performed Masson's trichrome staining. The test showed a reduction of the cKO alveolar bone mass and collagen fibers in the cKO PDL (Figure 2A). Our representative images of polarized light revealed decreased and disorganized collagen fibers in the cKO PDL (Figure 2B). To elucidate the cell fate of mesenchymal progenitor cells in postnatal alveolar bone formation, we examined the biological features of Gli1^*Lin*^ cells in the early cKO mice using cell lineage tracing techniques (i.e., removing *Tgf*β*r2* and activating the tdTomato fluorescent protein in Gli1^Lin^ PDL cells at the same time). At P6, there were few tdTomato+ cells in PDL area and in adjacent bone marrow areas after 24-h induction (Supplementary Figure 2A). By P28, there were numerous tdTomato+ cells throughout the entire PDL and alveolar bone, indicating a great contribution of the Gli1^Lin^ PDL progenitor cells to PDL and alveolar bone development during the period from P5 to P28 (Figure 2C, *left panel*). On the other hand, removing *Tgf*β*r2* in the Gli1^Lin^ cells led to a significant reduction in the number of tdTomato+ PDL cells (Figure 2C, *right panels*; Figure 2G, *left, P* = 0.0238, *n* = 3).

To study the underlying molecular mechanism, we examined expression levels of key molecules essential for PDL and alveolar bone formation. The immunostaining images showed a significant reduction of PCNA+ cells in the cKO PDL in comparison with control group (Figures 2D,G, *right, P* = 0.0259, *n* = 5), supporting the key role of TGFβ signaling in maintaining the proliferation rate of mesenchymal cells in PDL. Next, we analyzed the RNA expression level of β-catenin (a critical factor for differentiating of mesenchymal cells into osteoblasts) using RNAscope technique. This expression was sharply decreased in the early cKO PDL, supporting the likely role of TGFβ signaling in regulation of Wnt signaling (Figure 2E, *right panel*). Finally, we studied expression profiles of Periostin (a key matrix protein essential for PDL function) (Rios et al., 2005, 2008) at both RNA and protein levels. The levels were greatly reduced in cKO PDL (Figure 2F, *right panels*), suggesting the positive regulation of Periostin by TGFβ signaling in maintaining PDL homeostasis (Figure 2F).

### TGFβ Signaling Regulates Osteogenic Differentiation of PDL Progenitor Cells

**Figure 3:**
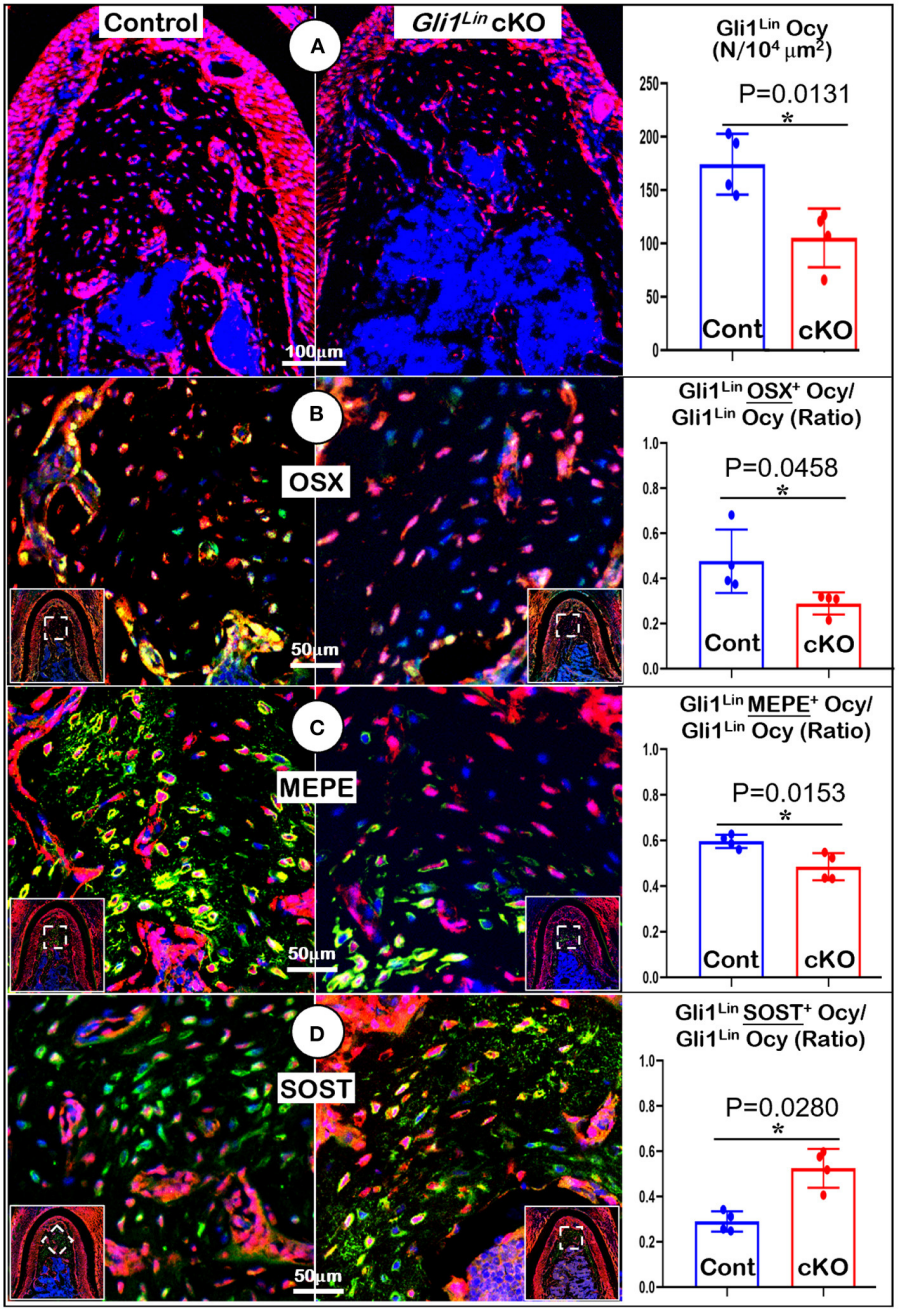
Molecular analyses of mandibles from Gli1^Lin^ *Tgf*β*r2* conditional knockout (cKO) mice **(A)** The confocal images revealed a great reduction in bone volume and number of Gli1^lin^ Ocy in cKO alveolar bone, which is statistically significant from the control (*right panel*); **(B)** The OSX immunostaining images showed a significant decrease in the raito of OSX^+^-Gli1^Lin^ Ocy to the total Gli1^lin^ Ocy; **(C**) The confocal images of MEPE immunostainings showed a significant reduction in the raito of MEPE^+^-Gli1^Lin^ Ocy to the total Gli1^lin^ Ocy in the cKO alveolar bone (*right panel*); **(D)** The confocal images of SOST immunostainings showed an increase in the raito of SOST^+^-Gli1^Lin^ Ocy to the total Gli1^Lin^ Ocy in the cKO alveolar bone, which was statistically significant from the control (*right panel*). *n* =4. *P < *0.05*; Ocy, osteocytes.

To understand the molecular mechanisms by which the abnormal alveolar bone occurred in early cKO mice, we examined expression levels of various bone markers. Markers were examined in the tracing background using immunostaining confocal techniques. Our data showed a significant reduction in tdTomato+ osteocyte numbers (Figure 3A, *P* = 0.0131, *n* = 4) and the ratio of Gli1^Lin^ osteocytes/total osteocytes (Supplementary Figure 2C, *right, P* = 0.0255, *n* = 4) in the cKO alveolar bone. The expressions of OSX (a transcriptional molecule essential for osteogenesis) (Zhou et al., 2010) were significantly decreased, which is reflected by the ratio of OSX^+^-Gli1^Lin^ osteocytes vs. total Gli1^Lin^ osteocytes in the cKO alveolar bone (Figure 3B, *P* = 0.0458, *n* = 4). We also showed a significant reduction in the MEPE levels of cKO Gli1^Lin^ osteocytes (Figure 3C, *P* = 0.0153, *n* = 4). On the other hand, there was a drastic increase of SOST, an osteocyte marker and potent inhibitor of Wnt signaling (Balemans et al., 2001) in the early cKO group. The ratio of SOST^+^-Gli1^Lin^ osteocytes vs. total Gli1^Lin^ osteocytes was significantly increased in early cKO mice compared to the control (Figure 3D, *P* = 0.0280, *n* = 4). We then performed TRAP staining to exclude the potential impact of removing *Tgf*β*r2* in Gli1^Lin^ PDL cells on osteoclast lineage cells. The staining showed no significant difference between the cKO mice and control (Supplementary Figure 3A, *P* = 0.0975, *n* = 4).

Collectively, the above data support the essential role of TGFβ signaling in controlling alveolar bone formation via regulation of Gli1^Lin^ PDL progenitor cell proliferation and differentiation.

### TGFβ Signaling Has a Moderate Effect on Osteoblasts

**Figure 4:**
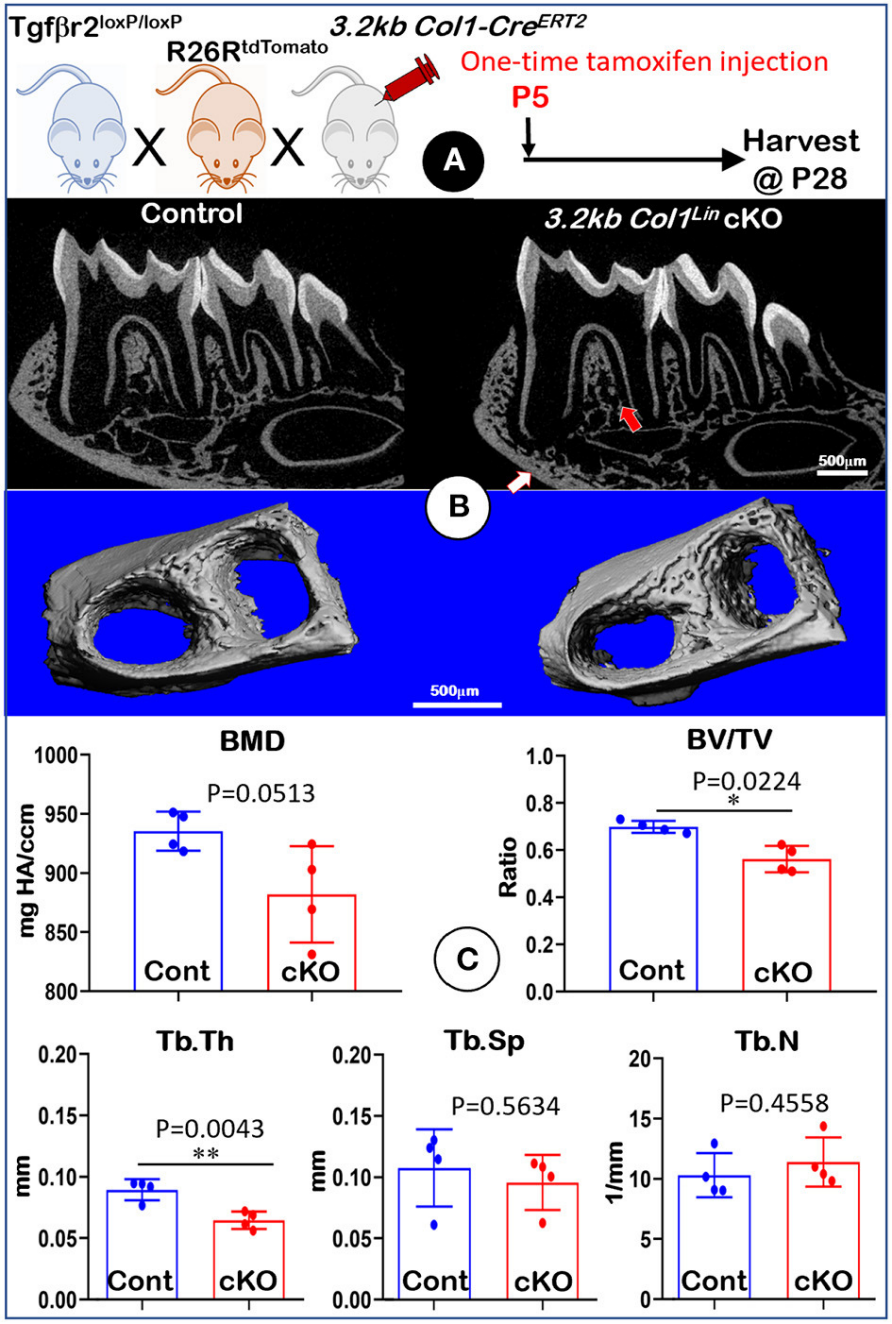
Micro-computed tomography (μCT) analyses of mandibles from *3.2 kb Col1*^Lin^ *Tgf*β*r2* conditional knockout (cKO) mice. **(A)** A schematic illustration of the generation of the *3.2 kb Col1-Cre*^*ERT*2^ induced *Tgf*β*r2* mice in the R26R^tdTomato^ tracing background. A one-time tamoxifen injection was administered at P5, and harvested at P28. **(B)** The cKO mice showed a mild decrease of bone mass at both trabecular (red arrow) and cortical (white arrow) regions. **(C)** Quantitative data based on the trabecular bone showed a reduction in bone mineral density (BMD) with no statistical significance (*upper left*), although there was a significant reduction in bone volume fraction (BV/TV) (*upper right*) and trabecular thickness (Tb. Th) (*lower left*). Of note, there was no apparent change in trabecular separation (Tb.Sp) (*lower middle*) and trabecular number (Tb.N) (*lower right*). *n* = 4, *P < *0.05*; **P < *0.01*.

To investigate the effect of TGFβ signaling on osteoblasts, we generated *Tgf*β*r2* cKO mice under the control of *3.2 kb Col1-Cre*^*ERT*2^ (Figure 4A). Similar to the *Gli1*^Lin^ *Tgf*β*r2* cKO mice, removing *Tgf*β*r2* in the 3.2 kb Col1^Lin^ cells had no apparent effects on the overall hindlimb structure (Supplementary Figure 4). Unlike *Gli1*^Lin^ *Tgfbr2* cKO mice, μCT data showed mild changes in alveolar bone of *3.2 kb Col1*^Lin^ *Tgf*β*r2* cKO mice (Figure 4B, *right panels, arrows*). Our representative μCT images showed a mild decrease in BMD (Figure 4C, *upper left, P* = 0.0513, *n* = 4) and the BV/TV of trabecular bone (Figure 4C, *upper left, P* = 0.0224, *n* = 4) in the late cKO mice. There was also a reduction of Tb.Th in cKO mice (Figure 4C, *lower left, P* = 0.0043, *n* = 4) with no statistic changes in Tb.Sp (Figure 4C, *P* = 0.5634, *lower middle, n* = 4) or Tb.N (Figure 4C, *lower right, P* = 0.4558, *n* = 4). Altogether, disruption of TGFβ signaling in osteoblasts led to a mild reduction in alveolar bone mass and mineral density.

**Figure 5:**
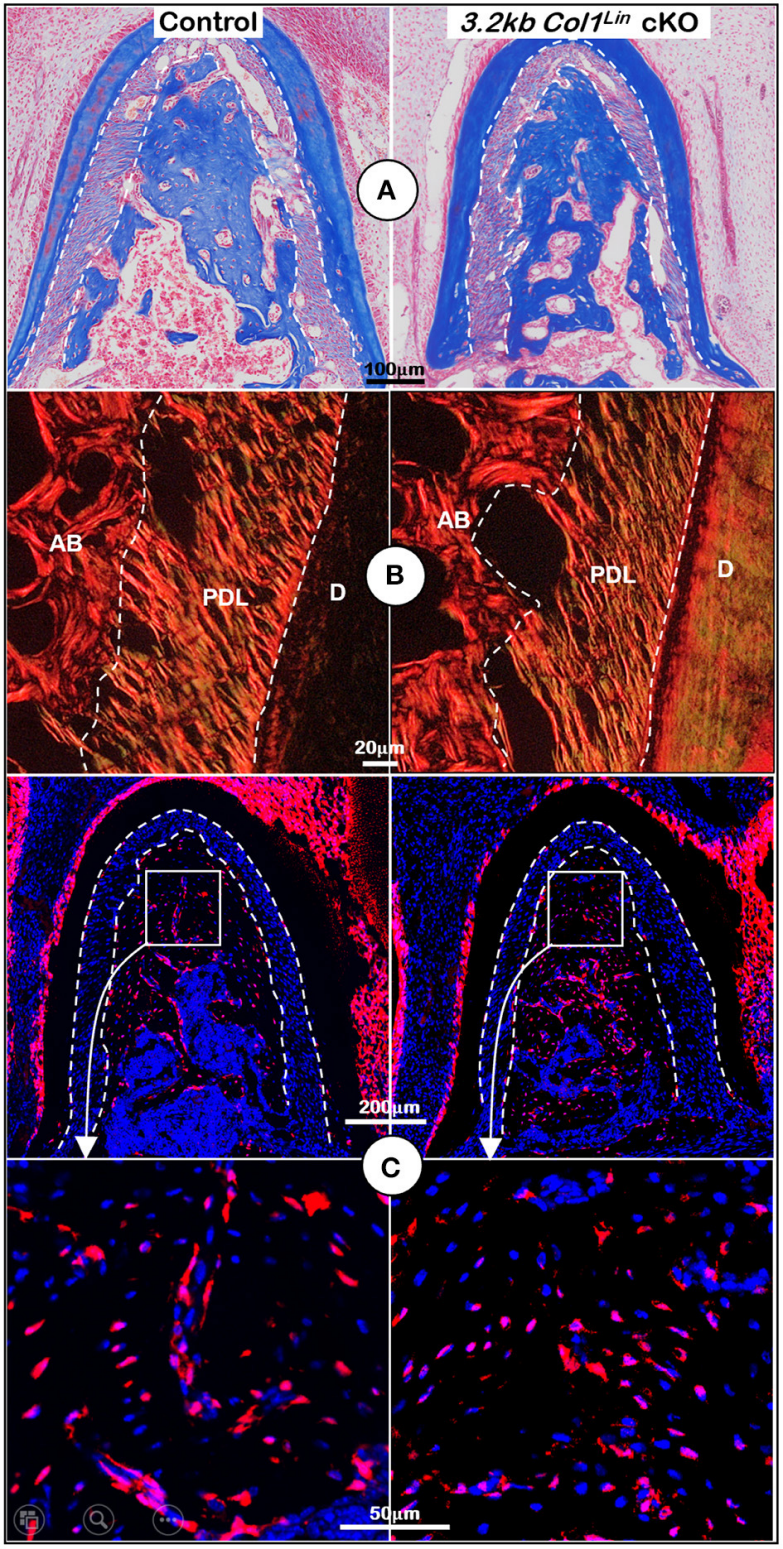
Morphological analyses of mandibles from *3.2 kb Col1*^Lin^ *Tgf*β*r2* conditional knockout (cKO) mice at the cellular level. **(A)** Masson's trichrome staining showed no apparent change of alveolar bone mass in the cKO mice (*right*). **(B)** Polarized light images displayed a similar collagen fiber distribution of periodontal ligament (PDL) in the control and cKO group (*right*). **(C)** The 3.2 kb Col1^Lin^ tracing images revealed largely similar red cell numbers in both the control and the cKO alveolar bone. AB, alveolar bone; PDL, periodontal ligament; D, dentin.

**Figure 6:**
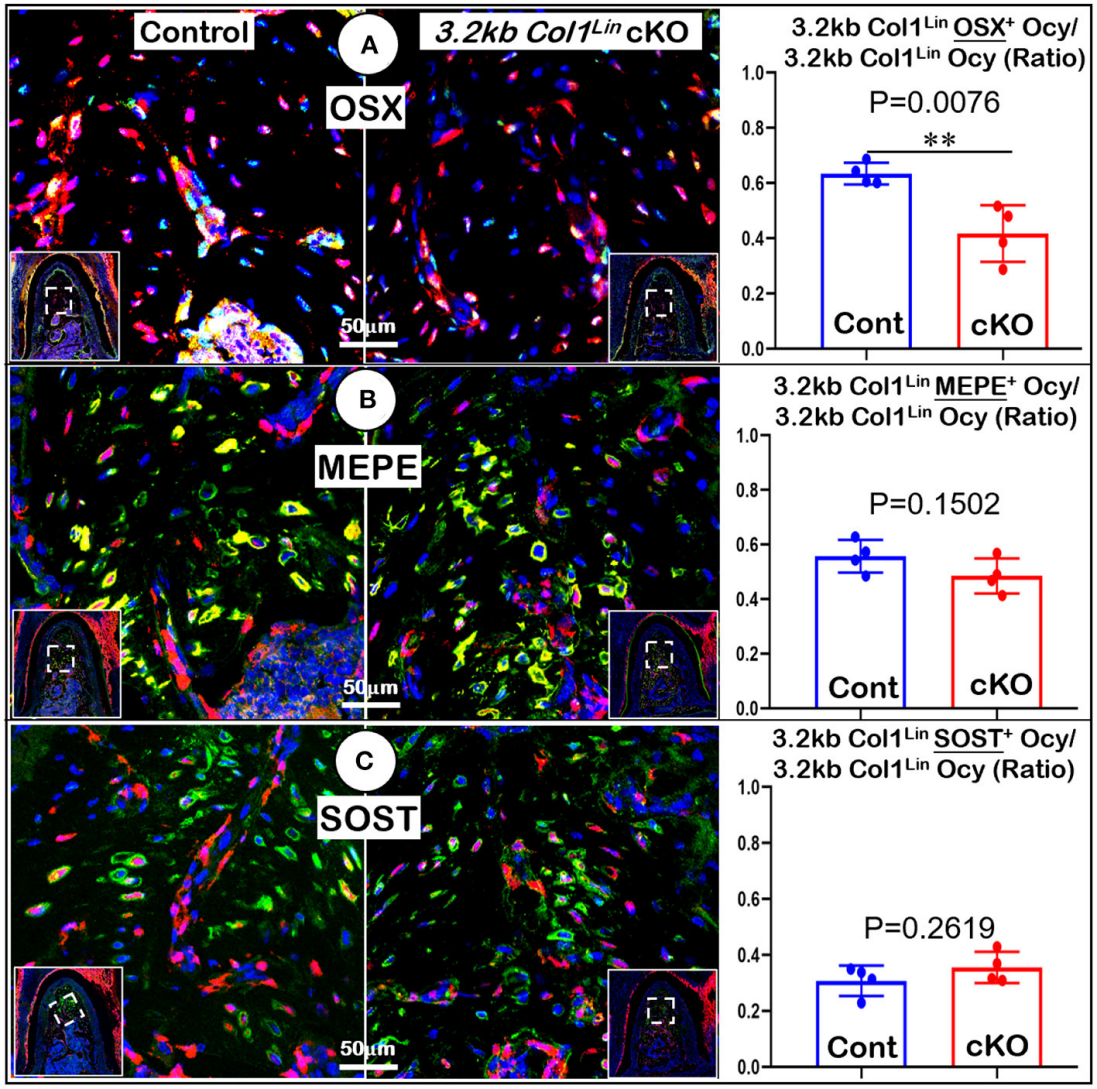
Molecular analyses of mandibles from *3.2 kb Col1*^Lin^ *Tgf*β*r2* conditional knockout (cKO) mice. **(A)** The confocal images of OSX immunostainings revealed a decrease in the raito of OSX^+^-3.2 kb Col1^Lin^ Ocy to the total 3.2 kb Col1^Lin^ Ocy in cKO alveolar bone, which was statistically significant from the control (*right panel*); **(B)** No apparent change was observed in the MEPE expression of cKO mice; and **(C)** The images of SOST immunostainings showed no apparent differences in signals between the cKO and control groups. *n* = 4. **P < *0.01*; Ocy, osteocytes.

To examine the impact of TGFβ signaling in late cKO mice at the cellular level, we performed Masson's trichrome staining. Our staining results showed no apparent changes in the cKO alveolar bone mass or in the PDL collagen fibers (Figure 5A). The representative images of polarized light revealed a distribution profile of collagen fibers in the cKO PDL, which was similar to that of the control group (Figure 5B). Our cell lineage tracing analyses showed no apparent change in tdTomato+ bone cell numbers or cell distribution pattern in cKO mice (Figure 5C, *right panels*; Supplementary Figure 2C, *left, P* = 0.8491, *n* = 4). We also examined the expression levels of several markers essential for bone function. The immunostaining showed a moderate but significant reduction in the expression ratio of OSX^+^-3.2 kb Col1^Lin^ osteocytes vs. total 3.2 kb Col1^Lin^ osteocytes in the cKO alveolar bone in comparison with control (Figure 6A; *P* = 0.0076, *n* = 4). However, removing *Tgf*β*r2* in the *3.2 kb Col1*^Lin^ cells had little impact on the expression ratio of osteocyte markers such as MEPE (Figure 6B, *P* = 0.1502, *n* = 4) or SOST (Figure 6C, *P* = 0.2619, *n* = 4).

## Discussion

TGFβ signaling plays an essential role in cell proliferation and differentiation of osteogenic cells during both intramembrane and endochondral bone formation. However, the role of TGFβ signaling during mandibular bone development remains largely unclear. In this study, we used inducible Cre mouse lines to remove *Tgf*β*r2* in osteogenic progenitors (*Gli1-Cre*^*ERT*2/+^; *R26R*^*tdTomato*/+^; *Tgf*β*r2*^*flox*/*flox*^; *Early cKO*) and osteoblasts (*3.2 kb Col1-Cre*^*ERT*2/+^; *R26R*^*tdTomato*/+^; *Tgf*β*r2*^*flox*/*flox*^; *Late cKO*), respectively. We aimed to provide a better understanding of the roles played by TGFβ signaling during postnatal alveolar bone formation. We used multiple techniques such as radiography, μCT, Masson's trichrome staining, and immunostaining combined with cell lineage tracing methods. Our comprehensive analyses showed drastic defects in the PDL and alveolar bone of early cKO mice but a moderate alveolar bone phenotype in late cKO mice. Our data support the essential role of *Tgf*β*r2* in osteogenic PDL cells during early alveolar bone development with limited impact on later osteogenesis.

The quantitative μCT analyses in our study showed that ablation of *Tgf*β*r2* in Gli1^Lin^ cells led to a significant reduction in alveolar bone volume and mineralization. This outcome was likely caused by a defect that occurred in PDL progenitors based on the following three pieces of information. (1) Molecular immunostaining and cell lineage tracing data revealed a significant reduction of PCNA+ and tdTomato+ PDL cells in the cKO mice; (2) The cell lineage tracing data showed a significant reduction of tdTomato+ bone cell numbers. (3) Masson's trichrome staining and polarized light images displayed defects in PDL and alveolar bone matrices along with a sharp decrease in three key molecules (Periostin in PDL, OSX, and MEPE in alveolar bone). In fact, the current findings of TGFβ signaling controlling cell proliferation and osteoblast differentiation agree with its roles in craniofacial bone and long bone (Sasaki et al., 2006; Peters et al., 2017).

Further mechanism studies using the RNAscope assay and immunostaining analyses showed that TGFβ signaling likely affected Wnt-β-catenin signaling, as shown by a sharp reduction in β-catenin mRNA within early cKO PDL and an increase in SOST (a potent inhibitor of Wnt signaling) within alveolar bone. Currently, we do not know whether there is a direct or indirect connection between the β-catenin in PDL and SOST inside alveolar bone. However, our previous studies demonstrated that removing *Periostin* in PDL led to severe defects in both PDL and alveolar bone; further deletion of sclerostin in osteocytes or applications of SOST- neutralized antibodies greatly improved both PDL and alveolar bone phenotypes via an interaction between Sharpey's fibers and osteocyte dendrites (Ren et al., 2015). TGFβ signaling may indirectly regulate Sharpey's fibers, a critical bridge between PDL and alveolar bone cells. This idea needs to be part of future studies.

Nevertheless, *Gli1-Cre*^*ERT*2^ was not only activated in PDL cells but also in cells from bone marrow, as shown from cell lineage tracing date in the present study and previous studies (Feng et al., 2017; Hosoya et al., 2020; Yi et al., 2021). To date, no specific markers could be used to discern differences between PDL-derived and bone-marrow-derived Gli1^Lin^ cells. However, the positive correlation between the Gli1^Lin^ PDL cell numbers and the Gli1^Lin^ alveolar bone cell numbers within the control support the contribution of Gli1^Lin^ PDL progenitor cells for alveolar bone formation. The negative correlation between the Gli1^Lin^ PDL cell numbers and the Gli1^Lin^ alveolar bone cell numbers within the early cKO further support this hypothesis.

It is previously reported that loss of TGFβ signaling in osteoblasts had an indirect effect on osteoclasts by reducing the number of osteoclasts in both long bones and mandibles (Qiu et al., 2010; Wang et al., 2013). However, our study observed no significant differences in either late or early cKO mice compared to their respective control groups. Results were based on TRAP staining, excluding the indirect role of osteoclasts on the bone loss phenotype.

**Figure 7:**
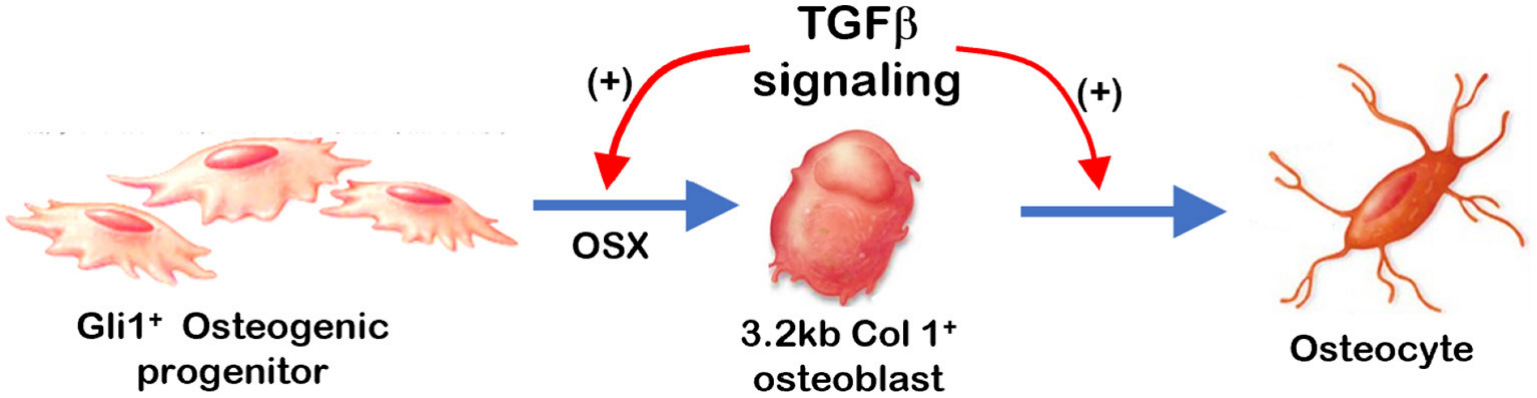
Summary: TGFβ signaling plays a stage-dependent role during mandible bone growth based on the loss of *Tgf*β*r2* in Gli1+ osteogenic progenitors and 3.2 kb Col1+ osteoblasts in the R26R^tdTomato^ tracing background, respectively. Removing *Tgf*β*r2* in the Gli1+ progenitor cells at early postnatal stage led to a significant bone loss and a decrease in OSX. Deletion of *Tgf*β*r2* in the 3.2 kb Col1+ osteoblasts at early postnatal stage result in mild changes in alveolar bone morphologies and bone volume.

Overall (Figure 7), removing *Tgf*β*r2* in the Gli1+ osteogenic progenitor cells led to significant alveolar bone loss as well as a decrease in OSX, β-catenin, PERIOSTIN, and MEPE. Conversely, there was a drastic increase in SOST (a potent inhibitor of WNT signaling). Deletion of *Tgf*β*r2* in 3.2 kb Col1+ osteoblasts resulted in mild changes in alveolar bone morphology and mild bone loss. Thus, we conclude that TGFβ signaling is essential for regulating the proliferation and differentiation of osteogenic progenitors during early postnatal alveolar bone formation.

## Data Availability Statement

The original contributions presented in the study are included in the article/Supplementary Material, further inquiries can be directed to the corresponding author/s.

## Ethics Statement

The animal study was reviewed and approved by Animal Care and Use Committees (IACUC) at Texas A&M University College of Dentistry and Sichuan University West China School of Stomatology.

## Author Contributions

CX contributed to the conception, design, data acquisition, analysis, interpretation, and also drafted and critically revised the manuscript. XX contributed to data acquisition and manuscript revision. YW and HZ contributed to data analysis and interpretation together with critically revising the manuscript. JW and JF contributed to conception, design, data analysis and interpretation for the project, and they drafted and critically revised the manuscript. All authors gave final approval and agreed to be accountable for all aspects of the work.

## Funding

This study was supported by grants from the National Institutes of Health (Grant Nos. DE025659, DE025014, and DE028291) to JF and from the National Natural Science Foundation of China (Grant Nos. 82071127 and 81700980) to JW.

## Conflict of Interest

The authors declare that the research was conducted in the absence of any commercial or financial relationships that could be construed as a potential conflict of interest.

## Publisher's Note

All claims expressed in this article are solely those of the authors and do not necessarily represent those of their affiliated organizations, or those of the publisher, the editors and the reviewers. Any product that may be evaluated in this article, or claim that may be made by its manufacturer, is not guaranteed or endorsed by the publisher.
