# Supplementary material for: TGF-Beta Receptor II Is Critical for Osteogenic Progenitor Cell Proliferation and Differentiation During Postnatal Alveolar Bone Formation
Source: Front Physiol. 2021 Sep 24;12:721775. doi: 10.3389/fphys.2021.721775 (PMC8497707; doi:10.3389/fphys.2021.721775)
Supplement: Supplementary file 1 [file Table_1.docx]

Supplemental Table 1: Primers for genotyping

| Gli-Cre^ERT2^ | Common: 5'-TCTGCCCACTCTTTGGGATG-3' |
| --- | --- |
|  | Mutant Forward: 5'-GCATTGCTGTCACTTGGTCG-3' |
|  | Wild-type Forward: 5'-ATGCGCTAGGGATGGAGTTG-3' |
| 3.2kb Col1-Cre^ERT2^ | Forward:5'-CCCGCAGAACCTGAAGATG-3' |
|  | Reverse: 5'-GACCCGGCAAAACAGGTAG-3' |
| R26R^Tdtomato^ | Forward: 5'-GGCATTAAAGCAGCGTATCC-3' |
|  | Reverse: 5'-CTGTTCCTGTACGGCATGG-3' |
| Tgfβr2 | Forward: 5'-TATGGACTGGCTGCTTTTGTATTC-3' |
|  | Reverse: 5'-TGGGGATAGAGGTAGAAAGACATA-3' |
