# Supplementary material for: TGF-Beta Receptor II Is Critical for Osteogenic Progenitor Cell Proliferation and Differentiation During Postnatal Alveolar Bone Formation
Source: Front Physiol. 2021 Sep 24;12:721775. doi: 10.3389/fphys.2021.721775 (PMC8497707; doi:10.3389/fphys.2021.721775)
Supplement: Supplementary file 2 [file Data_Sheet_1.docx]

**Fig. S1.** **Representative X-ray analyses of hindlimbs from *Gli1^Lin^ Tgfβr2* conditional knockout (cKO) mice.** There was no apparent differences in overall hindlimb length and mineral density between control (*left*) and the *Gli1-Cre^ERT2^* induced *Tgfβr2* cKO mice (*right*) by radiograph images.

**Fig. S2. Cell tracing analyses of Gli1^Lin^ and 3.2kb Col1^Lin^ cells in mandibles after 24-hour induction of Cre activity (P5-P6). (A)** Confocal images showed expression pattern of Gli1+ osteogenic progenitors and their descendants in the R26R^tdTomato^ tracing background (*upper panel*). Enlarged images revealed that only a few cells are activated at P6, some of which resided in PDL area and the others were located in bone marrow; white dot line outlined the shape of root furcation. *(lower panel*). (**B**) Confocal images showed expression pattern of 3.2kb Col1+ osteoblasts and their descendants in the R26R^tdTomato^ tracing background (*upper panel*). Enlarged images showed that 3.2kb Col1+ osteoblasts resided on the surface of the early-formed alveolar bone, and some of them formed bone; white dot line outlined the shape of root furcation. (*lower panel*). (**C**) Quantitative analyses showed that there was no significant change in the number of 3.2kb Col1+ ocy in alveolar bone of cKO (*left*); and the ratio of Gli1^Lin^ ocy/total ocy was significantly higher than 3.2kb Col1^Lin^ ocy/total ocy; and the ratio of Gli1^Lin^ ocy/total ocy was decreased due to conditional knockout of *Tgfβr2* (*right*). n=4, **P < 0.05*; ***P < 0.01.* Ocy=osteocytes; PDL=periodontal ligament; DP=dental pulp; BM=bone marrow*.*

**Fig S3. TRAP (tartrate-resistant acid phosphatase) assays from *Tgfβr2* conditional knockout (cKO) mice** (**A**) The TRAP staining images showed no significant differences between the control (*left*) and Gli1^Lin^ *Tgfβr2* cKO mice (*right*), n=4; and (**B**) the TRAP staining images showed no apparent differences between the control (*left*) and 3.2kb Col1^Lin^ *Tgfβr2* cKO mice (*right*, n=4).

**Fig S4.** **Representative X-ray analyses of hindlimbs from *3.2kb Col1^Lin^ Tgfβr2* conditional knockout (cKO) mice**. There was no apparent difference in overall hindlimb length and mineral density between control (*left*) and the *3.2kb Col1-Cre^ERT2^* induced *Tgfβr2* cKO mice (*right*) by radiograph images.
