# Supplementary figures and images for: TGF-Beta Receptor II Is Critical for Osteogenic Progenitor Cell Proliferation and Differentiation During Postnatal Alveolar Bone Formation
Source: Front Physiol. 2021 Sep 24;12:721775. doi: 10.3389/fphys.2021.721775 (PMC8497707; doi:10.3389/fphys.2021.721775)

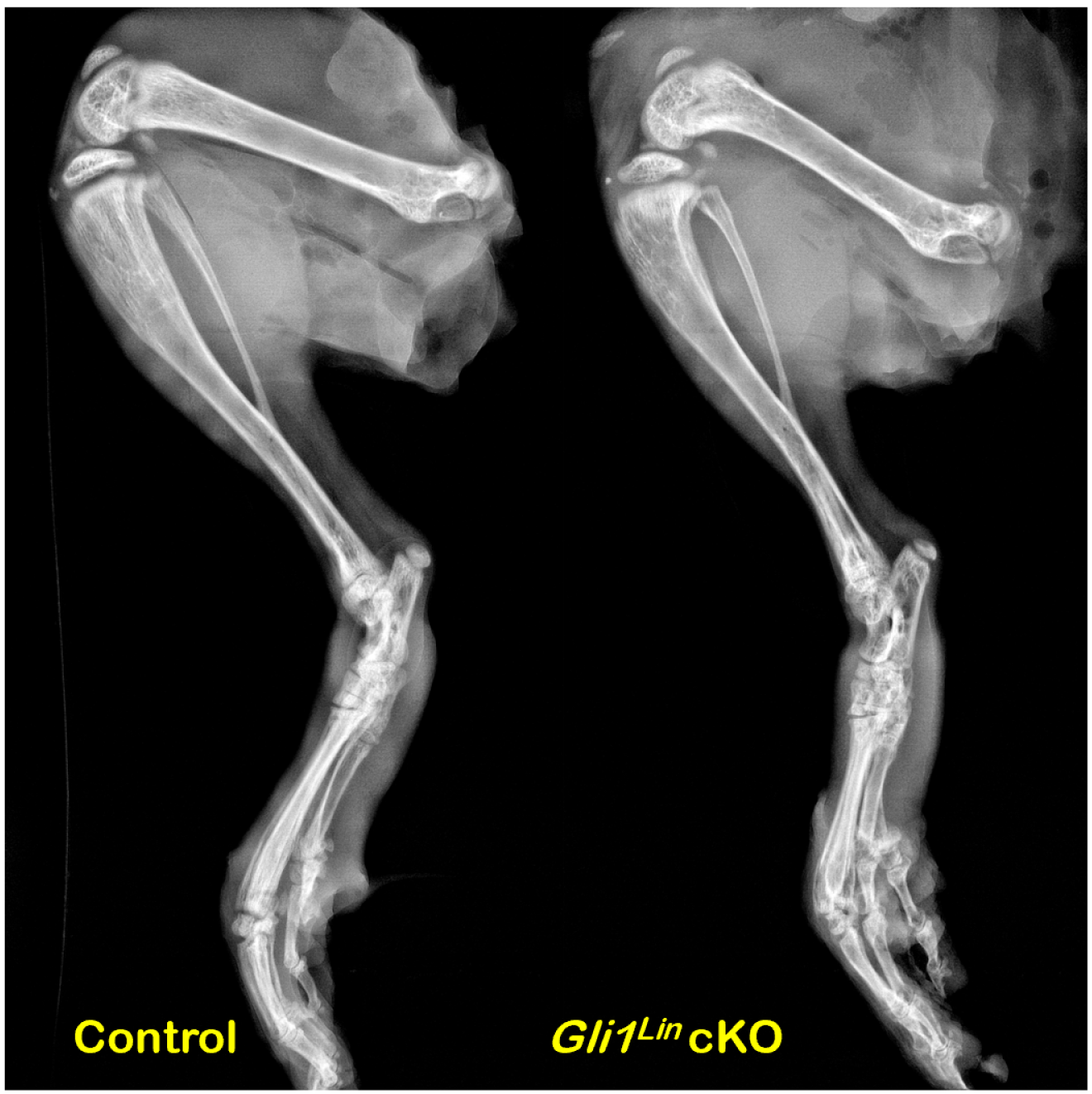

Supplement: Supplementary file 3 [file Image_1.TIF]

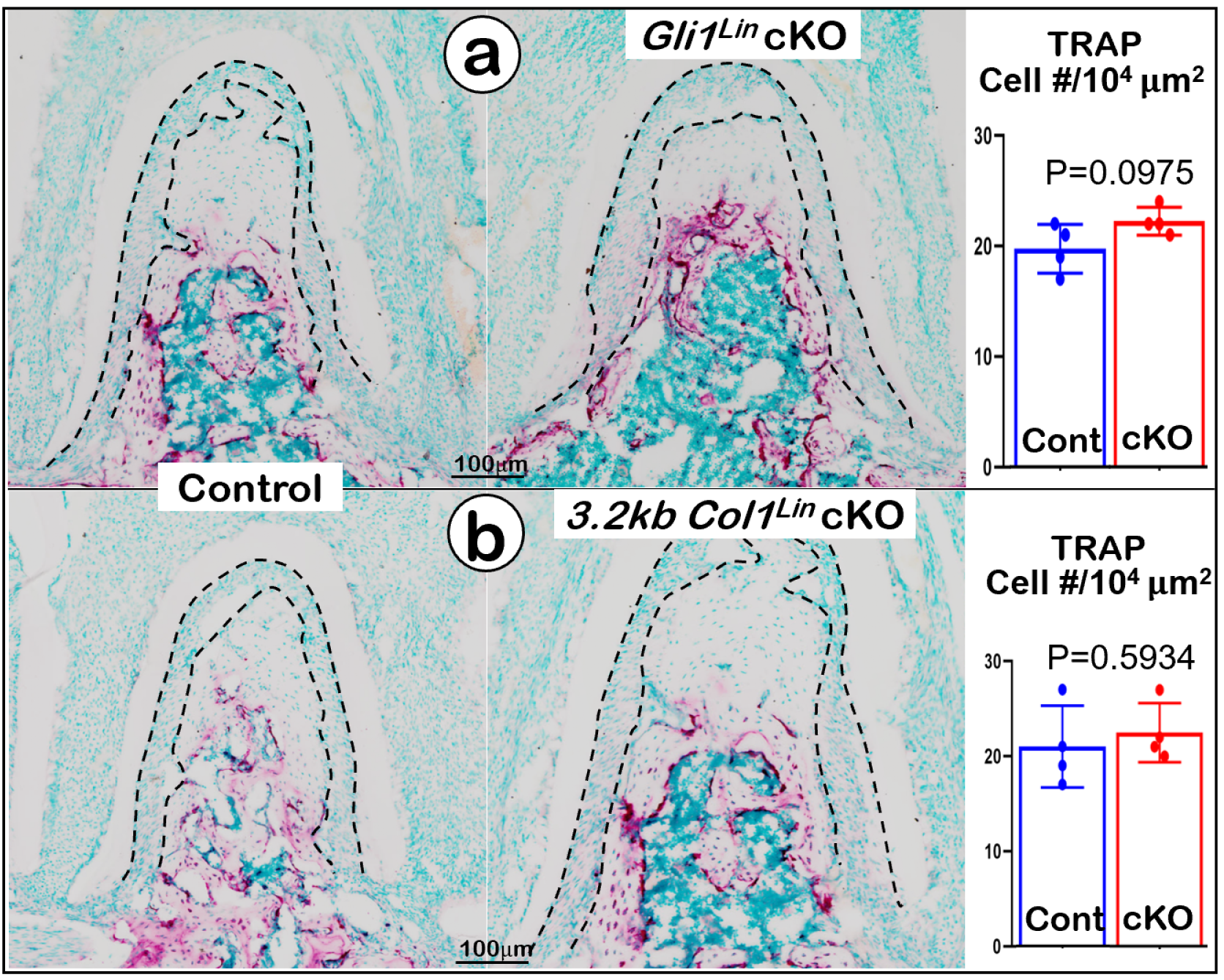

Supplement: Supplementary file 4 [file Image_2.TIF]

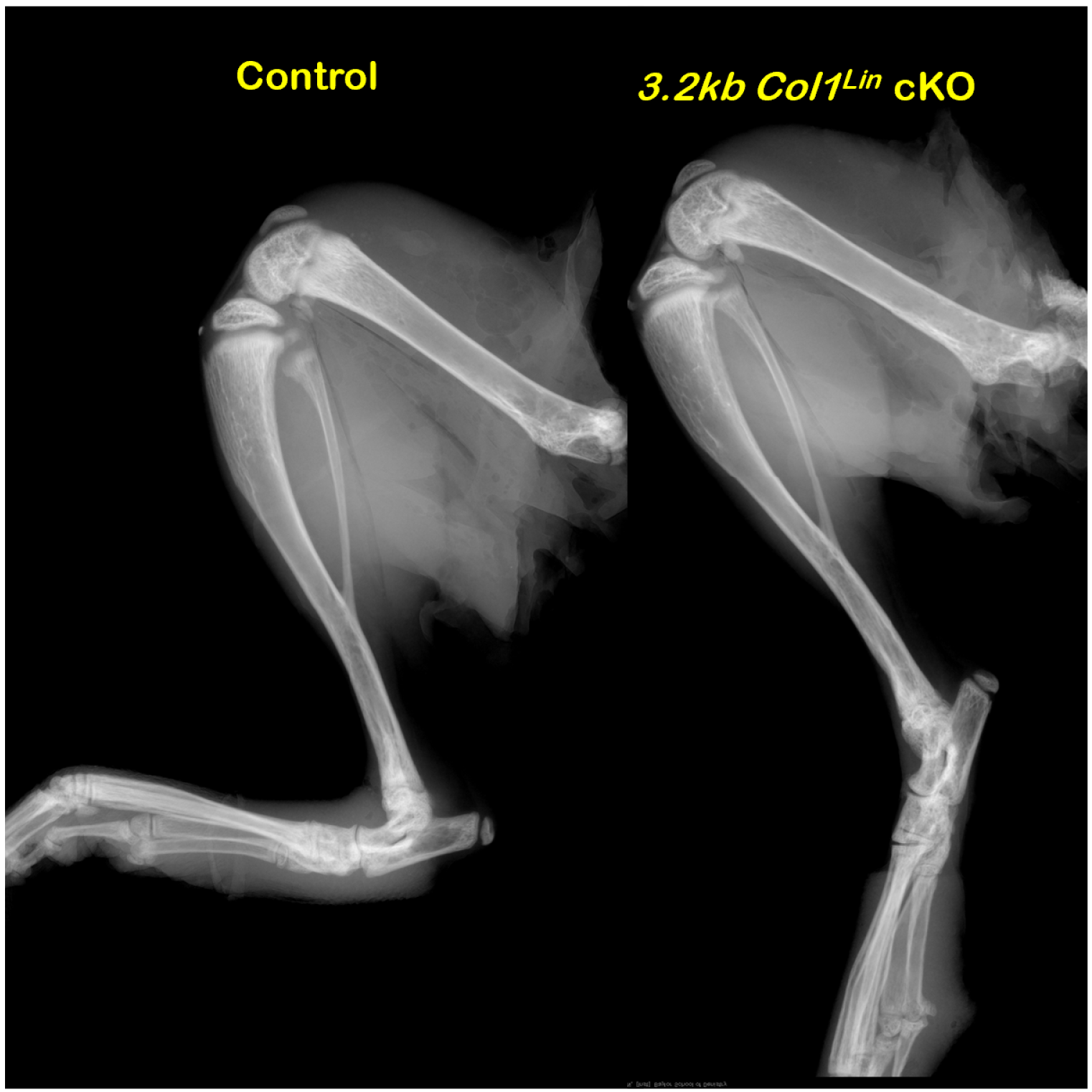

Supplement: Supplementary file 5 [file Image_3.TIF]

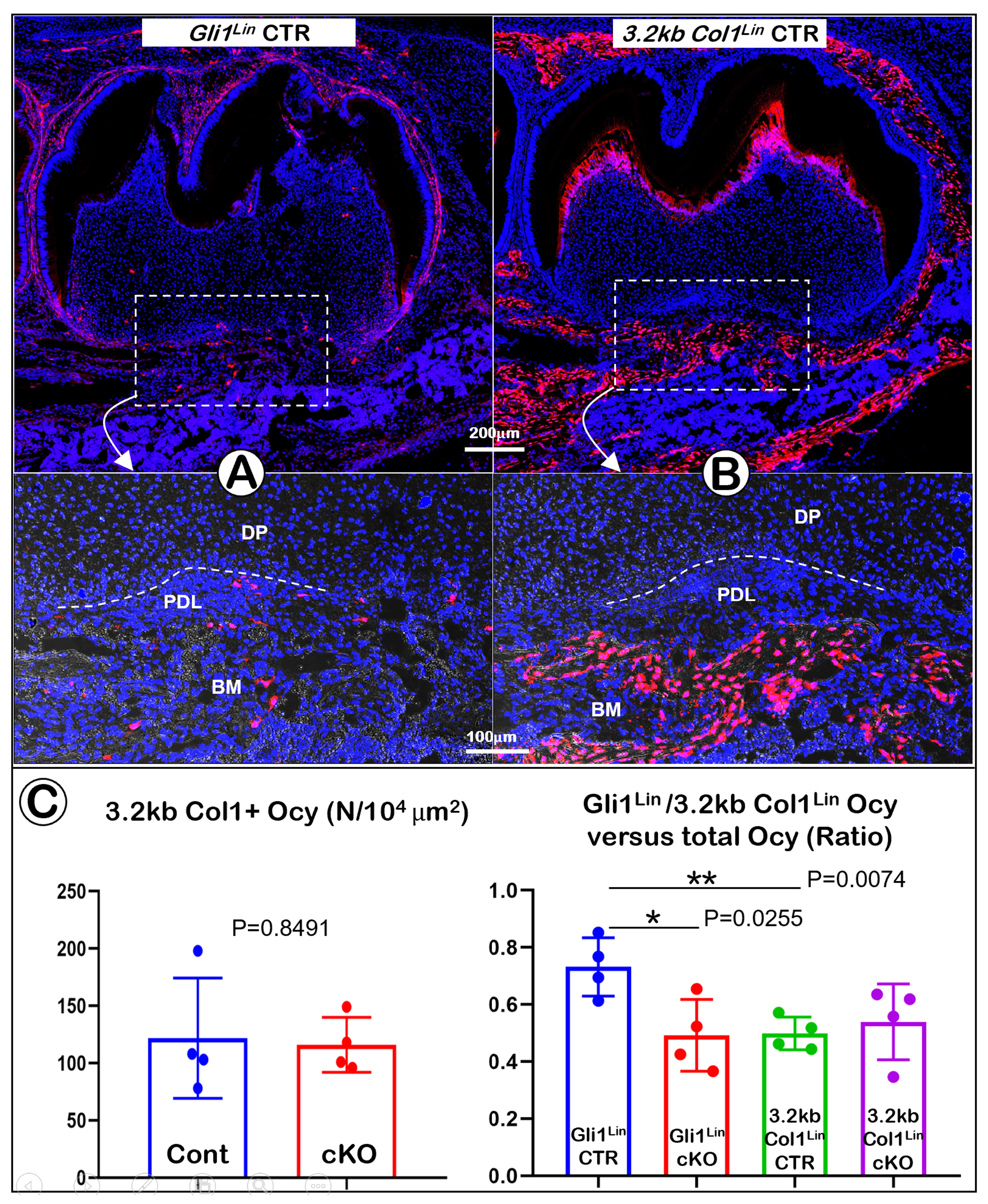

Supplement: Supplementary file 6 [file Image_4.JPEG]
